# Acoustic variation in alarm calls of Corvidae–effect of morphology, ecology and phylogeny

**DOI:** 10.1007/s10071-025-02000-w

**Published:** 2025-10-23

**Authors:** Eliška Kovářová, Pavel Linhart, Michaela Syrová, Jan Robovský, Nela Urbanová, Petr Veselý

**Affiliations:** Faculty of Science, University of South Bohemia, Branišovská 1760, 37005 České Budějovice, Czechia

**Keywords:** acoustic adaptation hypothesis, corvid, mobbing, peak frequency, harmonicity, call duration

## Abstract

Bioacoustic parameters of vocalization are generally well adapted to the environment that the species inhabits and are influenced by the morphology and life history of the species. These effects are frequently studied on bird songs; however, little is known about how ecology, phylogeny and morphology affect alarm calls. We investigated these factors in the crow family (Corvidae, Passeriformes) that exhibits extensive distribution around the world and is well known for their variable, conspicuous alarm calls. Using a free database of sounds (xeno-canto) as well as own recordings, we collected alarm calls of 66 species representing 21 out of 24 genera within the family. We measured four vocal characteristics, which showed high variability among the family and low mutual correlation – peak frequency, peak frequency change, harmonicity and call duration. The effect of body mass and preferred habitat openness of the species on these four parameters was tested using phylogenetic regression. We also assessed the strength of the phylogenetic signal. Peak frequency, peak frequency change and harmonicity were influenced by phylogeny, while the alarm call duration evolved independently of phylogeny. Body mass significantly affects the mean peak frequency with bigger species producing sounds of lower frequency. Additionally, the openness of the preferred habitat of the species significantly affects the maximum change in peak frequency. Forest species show less peak frequency fluctuation within the alarm call, probably because dense vegetation does not favour good transmission of highly modulated signals. We conclude, that features of corvid alarm calls are affected by similar drivers as e.g. bird song.

**Supplementary Information:**

The online version contains supplementary material available at 10.1007/s10071-025-02000-w.

## Introduction

Acoustic signalling is one of the most widely used forms of communication in birds, as calls evolved to be transmitted over large distances and address multiple receivers (Bradbury and Vehrencamp 1998). The efficacy of the acoustic signal, measured as successful detection and understanding at a required distance by the intended receiver and eliciting appropriate response, is affected by multiple internal as well as external factors (Bradbury and Vehrencamp 1998).

Birds have colonized a wide range of habitats that all have different sound transmission properties and constraints. The structure of a habitat, climatic conditions and ambient noise can all cause varying levels of sound attenuation, reverberation, overlap and degradation (Bradbury and Vehrencamp 1998). The acoustic adaptation hypothesis (AAH) suggests that the structure of birds’ acoustic signals has been shaped by selective pressures to maximize the effectiveness of transmission in their native environment (Morton 1975). This hypothesis has been extensively studied in a variety of habitats, both across species (Blumstein and Turner 2005; Mikula et al. 2020; Wiley 1991) and within species (Hunter Jr. and Krebs 1979; Nicholls and Goldizen 2006), but so far, no general consensus has been reached.

Morton (1975) was the first to suggest variability in transmission effectiveness of different frequencies in different habitats. In the forest habitat, he described a “frequency window” between 1.585 and 2.500 Hz where the signal is less attenuated compared to higher or lower frequencies and is, correspondingly, favoured by the local forest species. He also found that forest species tend to use more tonal signals. According to the AAH lower frequencies should overall be preferred in forests, since higher frequencies are generally more prone to attenuation in dense environments. In grasslands no similar window has been detected. Open habitats pose their own set of challenges. They are comparatively more unstable with frequently changing temperature, humidity, ambient noise levels and air turbulence caused by the wind. Morton’s results suggest that as a result of the instability of conditions, grassland species choose to rely on coding the information into modulated sounds and trills instead of a specific frequency. Naguib (2003) showed that there was a seasonal difference in the level of reverberation of a trill between a forest with and without foliage. The denser the forest, the more reverberation of the signal was detected. The level of reverberation also increased with the duration of the trill. Hunter Jr. and Krebs (1979) showed that Great tits (*Parus major*) from open woodlands had higher maximum frequency songs than tits from dense forests. The study sites were located in different countries all over Europe and still the songs were more similar to each other at sites with similar habitat type than at geographically closer sites. Additionally, forest Great tit songs were shown to be shorter with a lower number of notes than those of open habitat Great tits. Similar results were presented in the study by Nicholls and Goldizen (2006) on different populations of Australian satin bowerbirds (*Ptilonorhynchus violaceus*).

Ryan and Brenowitz (1985) suggested that the frequency window in forests is more likely caused by a relative absence of ambient noise rather than reduced attenuation. Indeed, ambient noise seems to play an important role in shaping the birds’ acoustic signal’s structure in songbirds and non-songbirds with innate vocalizations. For example, two species of *Pogoniulus* tinkerbirds sing at higher frequencies the closer they dwell to the coastline which has been suggested as an adaptation to avoid jamming with low frequency ocean noise (Sebastianelli et al. 2022). A similar trend can be observed in anthropogenic environments and ambient noise (Phillips et al. 2020; Slabbekoorn and Ripmeester 2008).

The effect of habitat on the acoustic signals’ structure was confirmed in further studies (Kirschel et al. 2009), but there were also studies identifying other evolutionarily more conserved factors as more important in shaping the structure (Mikula et al. 2020; Branch and Pravosudov 2020; Friis et al. 2021). Such studies often show that body size directly correlates with the size of the vocal apparatus of birds which in turn determines the frequencies the individual is able to produce most effectively. Body size therefore limits the bird’s ability to adaptively modify the frequency of its acoustic signals. The link between body size and various frequency characteristics of various species has been confirmed for example in Friis et al. 2021 for sound frequency, Martin et al. 2011 for minimum frequency and frequency of maximum amplitude and Mikula et al. 2020 for peak frequency). Other times, only limited support for AAH is stated because the role of phylogeny appears to be greater than that of ecological factors. Blumstein and Turner (2005)’s analysis of Australian bird songs initially appears to support the AAH with species from open habitats having shorter songs of higher frequency and greater bandwidth, but these results lose their significance after controlling for phylogenetic independence using the genus pair analysis. Although at the first sight the correlation between the bioacoustics parameters of the songs and the habitat type seemed clear, in this case phylogeny apparently plays a much larger role in shaping the sound structure. One possible explanation for the discrepancy between the results of various studies is that certain acoustic parameters can be phylogenetically conserved, while others can be ecologically plastic simultaneously. In herons (Ciconiiformes: Ardeidae) temporal characteristics, such as the number of syllables in a call, and frequency characteristics, such as the tonality of the call structure and fundamental frequency, are strongly linked to the phylogenetic history of the group. Closely related species share similarities in the morphology of their syrinx as well as calling behaviour which leads to similarities in the temporal acoustic parameters. On the other hand, peak frequency and frequency range seem to be more tailored to the habitat that species inhabit (McCracken and Sheldon 1997). While both Hunter Jr. and Krebs (1979) and Nicholls and Goldizen (2006) compared populations of the same species in their studies, McCracken and Sheldon (1997) compared different species, which might explain why the phylogeny influenced at least some of the bioacoustics parameters, e. g. the number of syllables and therefore also the whole call duration, more strongly than ecology.

Most studies have chosen songs as the focus of their examination for the AAH. Birds utilize songs mainly for territory defence and mate attraction, which both require the signal to travel over a long distance. Other signals like contact calls or alarm calls have been given remarkably less attention. These signals, however, do not need to be specifically designed for long range communication as they typically target close by individuals like flock or family members. In some cases, mobbing calls may be addressed to multiple other individuals including individuals outside their social group or heterospecifics (Griesser and Ekman 2004; Randler 2006; Klimšová and Policht 2015), which should favour acoustic adaptations for long distance propagation, and therefore follow the AAH. Although Friis et al. (2021) found no evidence of the AAH, they did find out that body size had a greater influence on songs than on contact calls of a number of passerine species. Martin et al. (2011) carried out a pairwise comparison between songs and distress calls (produced after the individual has already been captured by a predator) of 38 species of passerines and hummingbirds and concluded that songs had significantly lower frequencies of maximum amplitude than distress calls. Ecological factors seem to influence songs and other kinds of signals in different ways. Both contact calls and alarm calls are signals used mainly over short distance addressing flock and family members (Marler 2004; Griesser and Ekman 2004; Sridhar et al. 2009). Certain types of alarm calls seem to even be specifically designed to not carry well over a longer distance. E.g. the “seeet” alarm call is a special variation of the general flight alarm call. It is a call of a very high frequency and a very narrow frequency range with gradual onset and offset used by many small European passerines (Marler 1955). This structure might either prevent the predator from hearing it at all because of its insufficient hearing sensitivity to such high frequencies as was show in the study by Klump et al. (1986) with the Great tit (*Parus major*) and the Eurasian sparrowhawk (*Accipiter nisus*) or at least make it more difficult to localize the caller (Jones and Hill 2001). Mobbing (or scolding) alarm calls’ purpose, on the other hand, is to recruit as many individuals from the caller’s surroundings as possible and it might therefore be beneficial, if the sound carries well over a longer distance. To this day it is not clear what the main purpose of distress calls is and to whom they tend to be addressed – whether to the predator in the immediate vicinity of the caller or perhaps to potential helpers further away (Magrath et al. 2015). Selection pressure on the bioacoustics structure might therefore depend quite heavily on the intended purpose of the vocalisation. Devoting more attention to signals other than songs could be helpful for a better understanding of the acoustic adaptation theory.

We have decided to test the AAH and other alternative factors possibly playing a role in the acoustic design of corvid alarm calls, such as the body size and phylogeny. The Corvidae family comprising 132 species divided into 24 genera (www.jboyd.net, Jønsson et al. 2016) is especially suitable for testing this hypothesis as the numerous corvid species vary in body size as well as types of preferred habitat openness significantly. Since they exhibit exceptional intelligence and adaptiveness, they occupy the majority of the world’s regions and biomes. The corvids’ ability to produce songs is not as well developed as in other passerines, but their vocal repertoire of other types of signals is extensive and diverse. They are especially well known for their variable, very conspicuous alarm calls, which often play an important role in ecosystems.

We were able to collect a representative sample of species across the entire family using a public database, xeno-canto (www.xeno-canto.org; a community-sourced, open-access online repository) which contains almost 1 million of recordings to date. We conducted a bioacoustics analysis of the recordings and tested the effect of preferred habitat openness, body size and phylogeny on several acoustic characteristics of corvid alarm calls.

We tested the following hypotheses:

- Peak frequency characteristics of corvid alarm calls rely on the ecology of the particular species rather than phylogeny explained by the acoustic adaptation hypothesis.
  o Large-bodied corvid species produce alarm calls of lower frequency than smaller ones.
  o Spectral characteristics in general (peak frequency, frequency changes, harmonicity) of corvid alarm calls are affected by habitat type and can be explained by acoustic adaptation hypothesis
- Temporal characteristics (call duration) of corvid alarm calls reflect phylogeny rather than the ecology of the species.

## Methods

### Study group

The Corvidae family is a monophyletic family comprising 132 species in 24 genera (www.jboyd.net; Jonsson et al. 2016). They can be found all over the world except for Antarctica and a few oceanic islands. They live in a wide range of habitats from open dry deserts to dense tropical rainforests (del Hoyo et al. 2009) and present a high interspecies variation in body size. The biggest representative of the Corvidae group is the Common raven (*Corvus corax*) with its weight of up to 2 kg, the smallest is the Dwarf jay (*Cyanolyca nanus*) weighing only 40–42 g (del Hoyo et al. 2009). Corvids’ ability to produce a typical bird song with harmonic structure is limited in contrast to other passerine groups. However, they make up for it with a wide range of other types of vocalizations that are used in a variety of ecological and social situations (Cramp and Brooks 1992; Anjos and Vielliard 1993; Ellis 2008; Laiolo et al. 2009). As corvids are often social species, their alarm calls tend to be quite conspicuous and may be even eavesdropped on by other species including mammals (Randler 2006; Klimšová and Policht 2015; Davídková et al. 2020).

### Source of recordings

The recordings we used in our analysis originated from two sources. The first source was the online open-source database xeno-canto (www.xeno-canto.org; for the list of recordings see Online Resource 5). The recordings in the database can be filtered based on a number of parameters including length, quality or type of sound. We focused on recordings of corvid species labelled as “alarm call” or “mobbing”. Recordings marked as “distress call” were not included. Filtering based on more detailed criteria, like the type of predator the alarm call is directed at, was not possible due to most recordings missing such metadata. It is often difficult to ascertain what the bird’s motivation for alarm production is in the field. The source of distress for the bird can even be the recorders themselves. The division into “alarm calls” and “mobbing calls” on xeno-canto often does not reflect the situation very accurately. Both types were included to broaden the dataset, but the original types were not kept separate. As for the quality of the recordings, we did not make use of the automatic quality filtering. Instead, we examined all of the available recordings and made the decision whether to include them or not, individually one by one.

The other source of data for our analysis were own alarm call recordings of species naturally occurring in Central Europe. We managed to collect recordings from six species: the Common rook *Corvus frugilegus*, the Carrion crow *Corvus corone*, the Common raven *Corvus corax*, the Western jackdaw *Coloeus monedula*, the Eurasian magpie *Pica pica* and the Eurasian jay *Garrulus glandarius*. Our own recordings were collected from June 2020 to July 2022 using the Marantz recording device (PMD661) and directional microphone Sennheiser ME67. Unlike xeno-canto recordings, these recordings included information about the circumstances that prompted the production of the alarm call. The stimulus eliciting the natural alarm call production was a mount of an avian predator, either the Northern goshawk (*Accipiter gentilis*) or the Eurasian eagle owl (*Bubo bubo*). The person with the recorder stayed at a minimum of 30 m away from the presented raptor mount and under the cover of vegetation or terrain to avoid attracting the focal bird ‘s attention.

### Bioacoustic analysis

All the recordings were processed in the Raven Pro bioacoustic software (K. Lisa Yang Center for Conservation Bioacoustics at the Cornell Lab of Ornithology 2024) before the analysis. Noise between 0 and 500 Hz was filtered out. Each element of the alarm call detected in a recording was cut precisely at the start and at the end of the sound. Each call therefore comprises exactly one element (see Online Resource 7).

In cases where the species regularly uses multiple acoustically different alarm call types, the recordings were divided into groups based on the type of alarm call. Different types of alarm call of the same species were thus available to be analysed separately. Corvids use a wide range of acoustic signals that differ in their structure and function. Sadly, the signal repertoire has not yet been described in detail for most species. The alarm call types were thus divided based on their overall acoustic distinctness to the human ear. This division was then confirmed for a subset of species using the similarity cluster analysis in the Luscinia software (version 2.16.10.29.01; Lachlan 2007). The types are not meant to describe the ecological function, only acoustic distinctness.

Luscinia software was used for the bioacoustic parameter analysis. Four parameters, out of the 179 available in Luscinia, that showed high variability (standard errors) among corvid species and low mutual correlation (non-significant Spearmann correlation test) at the same time were chosen for further investigation. The first parameter, peak frequency mean (1), was calculated as the mean of the peak frequencies over the 5 ms (ms) windows during the call. The second parameter, maximum peak frequency change (2) describes how much the peak frequency fluctuates with time. The software calculates the curve incline based on the peak frequency values within each section lasting 5 ms and then 5 such sections before and after it. A peak frequency change of 0 means that the peak frequency is stable throughout the whole element, negative values indicate dropping peak frequency, positive values indicate rising peak frequency. The highest value from all measured changes becomes the call-specific value used in statistical analyses. The third parameter, maximum harmonicity (3) defines how close the sound is to a harmonic model. Tonal calls score the highest while noise scores the lowest. It is calculated for every section of the element lasting 5 ms and the highest value is used. The fourth parameter, the length (4) is the duration of the whole element in seconds (s). The values of the four aforementioned parameters (for visual examples see Online Resource 7) were measured for each call. The mean value was calculated for all calls from the same recording (further referred to as “recording mean”). The mean value was then calculated for all recordings from the same species (further referred to as “species mean”). Different types of alarm calls of the same species were still kept separate. All values were log-transformed before conducting the statistical analyses.

The data for the bioacoustic analysis are available in the Online Resource 6.

### Morphological and ecological factors

Information about body mass and preferred habitat openness for all species included in the analysis was collected from literature (del Hoyo 2009). Body mass was defined as the average body weight in grams (g). If the weight in literature was stated as a range or separately for male and female, the mean value was calculated. Every species was assigned one of three preferred habitat openness categories based on the information in literature: open, closed or mixed. The “open” category represented species living mainly in deserts and steppes, while the “closed” category represented mainly forest species. Generalist species were put in the “mixed” category. Following these guidelines, 34 species in total were sorted into the forest category, 13 species into the open category and 19 species into the mixed the mixed category.

The dependency analysis of the chosen acoustic parameters on the morphological and ecological variables, i.e. the body mass and the preferred habitat openness with the phylogenetic correction was conducted in the R software (R Core Team (2024). Phylogenetic data were acquired from the Maximum Clade Credibility Tree created for Jønsson et al. (2016). Models were created using the *pgls* function from “caper” package for R (Orme et al. 2023). Statistical significance was tested using the *anova.pgls* function.

The data for testing the morphological and ecological factors are available in the Online Resource 6.

### Phylogenetic signal and reconstruction of continuous characters

The strength of the phylogenetic signal of individual traits was estimated by calculating Pagel’s lambda (λ), which allowsa more complex model of evolution with strong (λ = 1) to weak (λ = 0) phylogenetic covariation, and Blomberg’s *K*, which is a widely used metric to test the phylogenetic signal, using the phylosig function in the phylosignal package (Keck et al. 2016). Blomberg’s *K* allows us to determine if a trait carries a strong phylogenetic signal (K > 1), if it evolved under the Brownian model of evolution (K = 1), or if the trait underwent random diversification in related species and carries no phylogenetic signal (K = 0).

The Brownian motion model was selected for the calculation of Blomberg’s *K* for all the traits. Additionally, all the results were tested for statistical significance (*p* < 0.05). We used the Maximum Parsimony approach for the reconstruction of continuous characters implemented in Mesquite v3.81 (Maddison and Maddison 2023). All four inspected sound parameters were reconstructed as continuous characters with the squared change assumption. For the analysis we used the composite phylogenetic tree based predominantly on Jønsson et al. (2016), and partially also on Bonaccorso et al. (2010), Fernando and Townsend (2017), McCormack et al. (2010), and Song et al. (2018). The outgroups were selected based on the known phylogenetic relationships (Jønsson et al. 2016; McCullough et al. 2023) and the availability of recordings.

## Results

A total of 421 recordings were collected from the xeno-canto database and 123 recordings from the field. We managed to acquire at least one alarm call recording for 66 species of 21 genera, which constitutes 50% of all the corvid species.

### Phylogenetic signal

The values of Pagel’s Lambda and Blomberg’s *K* calculated for continuous characters indicate that the evolution of peak frequency, peak frequency change and harmonicity was driven to non-random diversification (see Table 1). Specifically, the peak frequency exhibited the strongest phylogenetic signal. These values suggest that traits deviate more from phylogeny than they would if they had evolved under the Brownian model of evolution. Conversely, the alarm call duration evolved independently from phylogeny and was driven by random diversification. Character reconstructions showed a complex pattern for every sound parameter, comprising many convergences, reversals, and a high disparity among closely related species (see Online Resources 1–4).

**Table 1:** A list of selected parameters of corvid alarm calls with their Pagel’s lambda and Blomberg’s *K* values together with their p values. Significant differences from the random Brownian model are highlighted in bold

Display	*λ*	p(λ)	*K*	*p(K)*
Peak frequency mean	**0.86**	1.5*e^−8^	**0.38**	0.02
Peak frequency change maximum	**0.48**	0.02	0.22	0.07
Duration	0.51	0.09	0.17	0.4
Harmonicity maximum	**0.77**	4.6*e^−5^	0.25	0.08

### Body mass

**Fig. 1:**
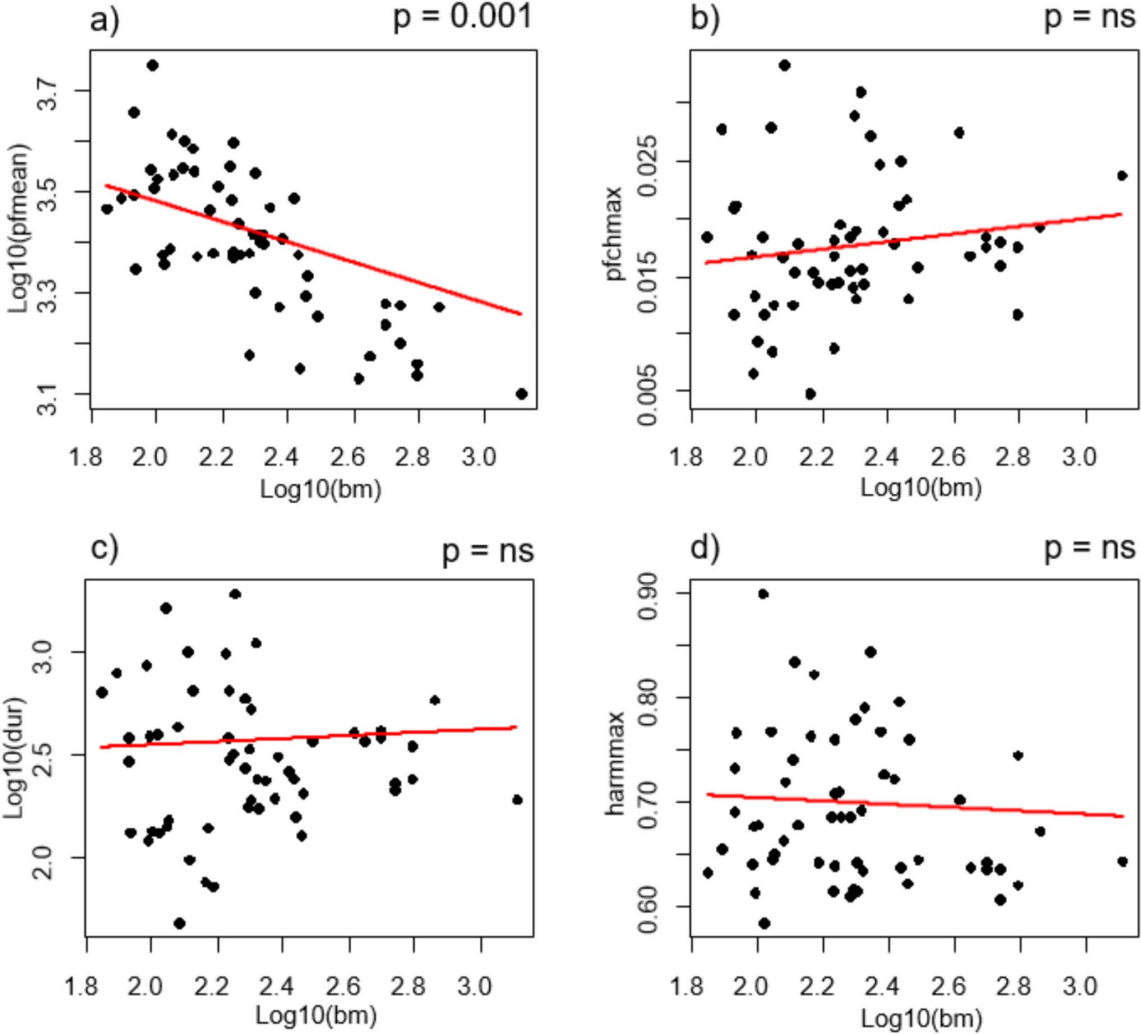
The relationship between **a**) peak frequency mean—log10(pfmean), **b**) peak frequency change maximum—pfchmax, **c**) duration – log10(dur) and **d**) harmonicity maximum—harmmax of corvid species alarm calls and their body mass log10bm. Both body mass and acoustic parameter values were transformed with decadic logarithm. The red line indicates the trend. The figure was created using R Statistical Software

Analyses taking into account the phylogenetic relationship of tested species revealed a significant relationship between body mass and peak frequency of the alarm call, (PGLS ANOVA; F_(55,1)_ = 11.40; λ = 0.9; p = 0.001) with species with larger body mass producing alarm calls of lower peak frequency (PGLS, λ = 0.9; β = −0.201; t = −3.377; p = 0.001; Fig. 1a). Body mass did not have a significant influence on variability of peak frequency change (PGLS ANOVA; F_(55,1)_ = 1.34; λ = 0; p = 0.252; Fig. 1b), duration (PGLS ANOVA; F_(55,1)_ = 0.13; λ = 1.0; p = 0.716; Fig. 1c) or harmonicity (PGLS ANOVA; F_(55,1)_ = 0.16; λ = 1; p = 0.690; Fig. 1d) of the alarm call.

### Preferred habitat openness

**Fig. 2:**
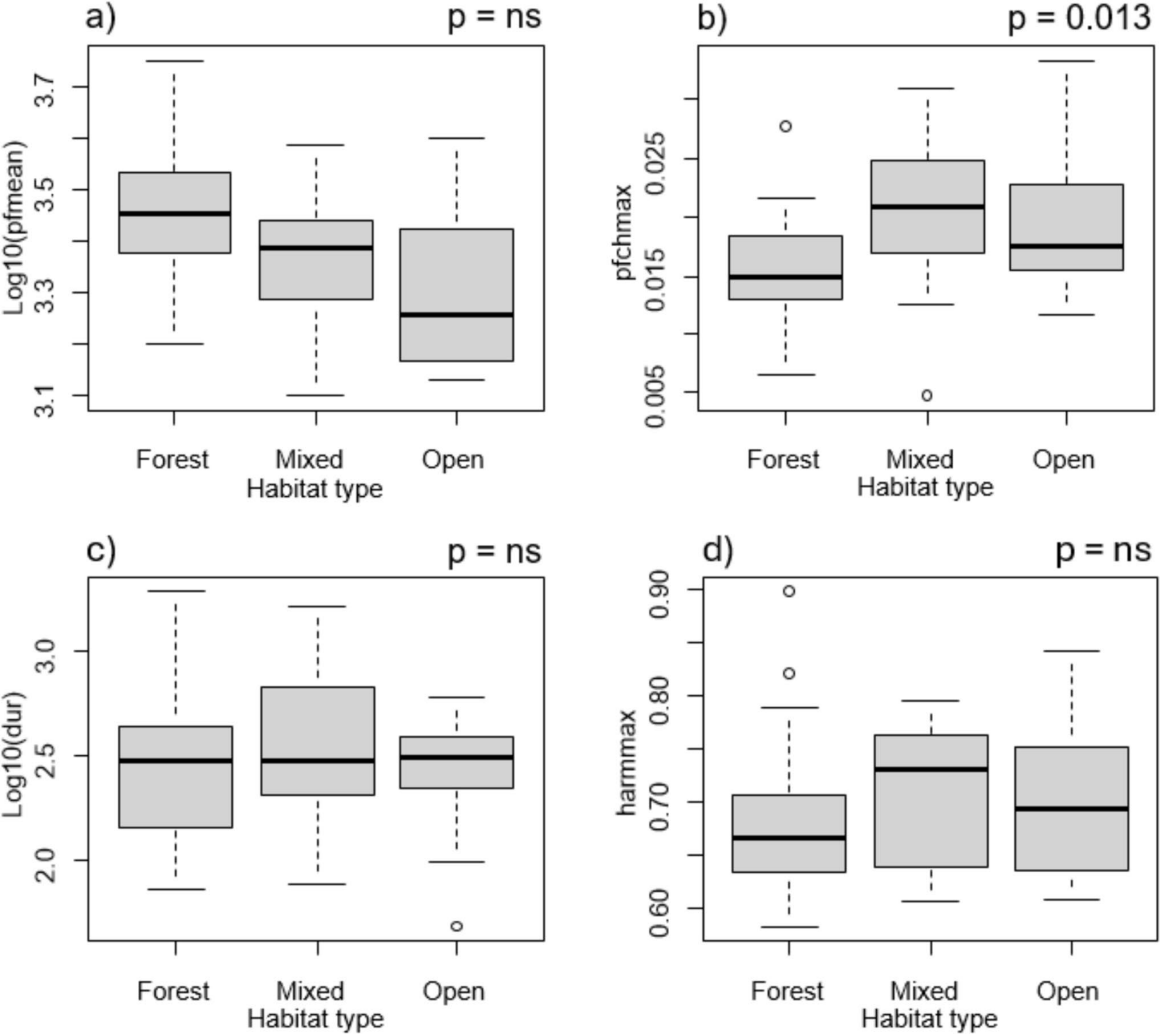
The relationship between **a**) peak frequency mean, **b**) peak frequency change maximum, **c**) duration, and **d**) harmonicity maximum of all the acquired corvid species and their preferred habitat openness. Acoustic parameter values are logarithmic. Habitats are divided into three categories according to vegetation density – forest, mixed and open. The figure was created using R Statistical Software

Preferred habitat openness significantly influenced the values of peak frequency change (PGLS ANOVA; F_(54,2)_ = 4.71; λ = 0; p = 0.013) with species living in enclosed forest habitats having lower rate of fluctuation in peak frequency than species from open habitats (PGLS; λ = 0; β = 0.004; t = 2.037; p = 0.047) or generalist species (PGLS; λ = 0; β = 0.005; t = 2,811; p = 0.007; Fig. 2b). Preferred habitat openness did not affect the peak frequency (PGLS ANOVA; F_(54,2)_ = 0.46; λ = 0.78; p = 0.633; Fig. 2a), duration (PGLS ANOVA; F_(54,2)_ = 0.26; λ = 1; p = 0.775; Fig. 2c) or harmonicity (PGLS ANOVA; F_(54,2)_ = 1.99; λ = 0.293; p = 0.146; Fig. 2d).

## Discussion

Different acoustic parameters of Corvidae alarm calls are influenced by phylogenetic relationships and ecological preferences to varying degrees. The acoustic adaptation hypothesis is, therefore, only partially supported by our data. The openness of the preferred habitat significantly affected only the peak frequency change with forest corvid species showing less frequency fluctuation in their alarm calls. The alternative hypotheses were also partially supported. Species with higher body mass produced alarm calls of significantly lower peak frequency. Phylogeny had a significant influence on the peak frequency, peak frequency change and harmonicity, while call duration evolved independently from phylogeny and is therefore, possibly, more dependent on the species’ ecology.

Our results show that peak frequency is the most phylogenetically influenced trait in corvids, perhaps due to some morphological constraints such as the body mass as indicated by our results and other related studies (Friis et al. 2021; Goller and Riede 2013; Martin et al. 2011; Mikula et al. 2020). Body mass has been shown to be closely linked to the phylogenetic history of bird species worldwide (Morales-Castilla et al. 2012). Larger-bodied bird species tend to produce calls of lower frequency regardless of the type and function of the vocalisation. With increasing body mass, the size of the syrinx increases, and species with a larger syrinx are capable of producing lower frequency calls more effectively than calls of higher frequency (Goller and Riede 2013; Ryan and Brenowitz 1985). Martin et al. (2011) specifically states that body size affects distress calls even stronger than songs in neotropical bird communities. Other studies described the same relationship between body mass and various frequency characteristics (Billings 2018; Laiolo and Rolando 2003a, b) On the other hand, the study by McCracken and Sheldon (1997) showed an association between body size and fundamental frequency. The peak frequency, however, changed with the structure of the species’ preferred habitat following the AAH. At least some avian lineages might thus be able to modify the peak frequency of their calls to better suit their environment.

No phylogenetic signal was detected in the alarm call duration, although associations between phylogeny and temporal characteristics including call duration have been reported in other studies, namely, in the oscine songs of North America (Wiley 1991), in heron calls (McCracken and Sheldon (1997) or in wood warbler flight calls (Farnsworth and Lovette 2008). Billings (2018) investigated mobbing calls of Corvidae and two other passerine families, and phylogenetic relationships among the species did not appear to be a strong predictor of the acoustic variability of their mobbing calls. Longer calls were instead associated with larger body mass. Laiolo and Rolando (2003b) also found a significant positive relationship between the corvid caw call duration and wing length which served as proxy for body mass. Our data, however, did not show any association between call duration and body mass or phylogenetic history. Call durations generally show high intraspecific variability (e.g. Zhang et al. 2022), which may limit the effect of the phylogenetic signal per se.

No significant difference in peak frequency of corvid species from closed and open habitats has been found. Even if the results were significant for our data, the trend as shown in Fig. 2 suggests higher, rather than lower peak frequency in forest corvids. Such a result is in contrast to the conclusions of other studies like Billings (2018), McCracken and Sheldon (1997) or Nicholls and Goldizen (2006) as well as for the maximum frequency measures in Hunter Jr. and Krebs (1979). Lower frequencies should withstand degradation in dense vegetation better than higher frequencies and should therefore be better suited for the dense forest environment. Corvid mobbing calls are very often addressed to multiple other individuals including individuals outside their social group or heterospecifics (Griesser and Ekman 2004; Randler 2006; Klimšová and Policht 2015) which should favour acoustic adaptations for long distance propagation. Our dataset supposedly includes flight alarm calls as well as mobbing calls (Wiley 1991).

The outcome of our analysis for peak frequency change is in line with the acoustic adaptation hypothesis. The fluctuation of peak frequency has been recognized as one of the determining features of distress calls (Aubin 1989). However, we found that forest corvid species use alarm calls with less frequency modulation compared to species from more open habitats. A delicate structure characterized by short element repetition periods or a lot of rapid frequency modulation is much more prone to degradation due to the density of the forest vegetation (Morton 1975; Wiley 1991; Naguib 2003; Nicholls and Goldizen 2006). Laiolo and Rolando (2003b) even reported the same trend of less frequency modulation in the forest habitats for the genus *Corvus*. The species in open landscapes are probably not forced to limit their peak frequencies, resulting in the preserved high level of modulation.

Morton (1975) reported in his study that forest bird species tend to use calls of pure tonal quality and, correspondingly, narrower frequency range more often than those living in more open habitats. However, corvid alarm calls are rarely tonal. Alarm calls of corvids usually include a wide frequency bandwidth. Tonal calls with narrow frequency bandwidth are found quite rarely in the corvids’ alarm repertoire (e.g. in choughs). Therefore, the variable we measured is defined on the scale harmonicity-noise rather than characterizing high tonality and narrow call frequency bandwidth. Our results did not show any association of harmonicity with either preferred habitat openness or body mass. Other similar studies also showed inconsistent picture. Aubin and Bremond (1991) argued that distress calls of starlings (*Sturnus vulgaris*) containing harmonic structures might spread better through dense vegetation than tonal calls. Wiley (1991) on the other hand, found no connection between the presence of harmonics in the songs of North American oscines and their preferred habitat. Therefore, it seems that concentration of sound energy to narrow frequency bandwidth is more important in the context of AAH than periodicity of sound.

## Conclusions

Overall, we found support for spectral characteristics of corvid alarm calls being affected by species ecology with some findings supporting AAH. We confirmed the expected relationship between body size and peak frequency. Surprisingly, we found neither strong phylogenetic signal nor a relationship to species ecology for call duration. These findings on alarm calls broadly correspond to those found for bird song, but seem to be less prominent, maybe because alarm calls do not necessarily need to be adapted for long-distance communication.

### Limitations and future plans

Xeno-canto as the source of data has been successfully utilized in numerous previous bioacoustic studies (Planqué and Vellinga 2008; Vellinga and Planqué 2015; Sarasa et al. 2017; Benedetti et al. 2018). Both experts and enthusiastic members of the public can contribute to the database which has helped it grow to the current almost 1 million recordings. This approach, however, also posed several challenges at the same time, like frequently missing important metadata or the fluctuating quality of recordings. As a result, our measurements could have potentially been influenced by factors impossible to account for, like the used recording device or weather conditions at the time of the recording. Additionally, a large portion of the recordings on xeno-canto is only available in the.mp3 format which is less suitable for the bioacoustic analyses than for example the.wav format.

Recordings of common synanthropic corvid species from temperate regions are much more prevalent in the database compared to rarer and more elusive species inhabiting dense tropical rainforests. Other species’ recordings are missing completely. In this study, even species with just a single available recording were included in the analyses to favour the interspecies variability at the cost of the intraspecies variability. Future research would greatly benefit from broadening the analysed dataset for each species. A greater number of species recordings could also potentially mitigate the unintended variability caused by different field recording methodology and other factors.

Although corvids represent a valuable study group due to their wide range of body sizes and ecological niches, it might be beneficial to first catalogue each species’ vocal repertoire in detail in order to better understand the ecological relevance of the different calls. Corvids use a variety of acoustic signals and even their alarm calls can significantly differ in their structure and function. Apart from the basic division into flight, mobbing and distress alarm calls, corvids are known to modify the structure of their alarm to encode additional information like the species of the predator and the level of threat it poses (Griesser 2008; Yorzinski and Vehrencamp 2009). The resulting extreme structure variability makes the bioacoustic comparison of alarm calls of different species very complicated.

## Supplementary Information

Below is the link to the electronic supplementary material.

Supplementary file1 (TIFF 334 kb)

Supplementary file2 (TIFF 343 kb)

Supplementary file3 (TIFF 343 kb)

Supplementary file4 (TIFF 333 kb)

Supplementary file5 (XLSX 102 kb)

Supplementary file6 (XLSX 19 kb)

Supplementary file7 (PNG 372 kb)

## Data Availability

Data is provided within the supplementary information files.
