# Supplementary figures and images for: Acoustic variation in alarm calls of Corvidae–effect of morphology, ecology and phylogeny
Source: Anim Cogn. 2025 Oct 23;28(1):83. doi: 10.1007/s10071-025-02000-w (PMC12549722; doi:10.1007/s10071-025-02000-w)

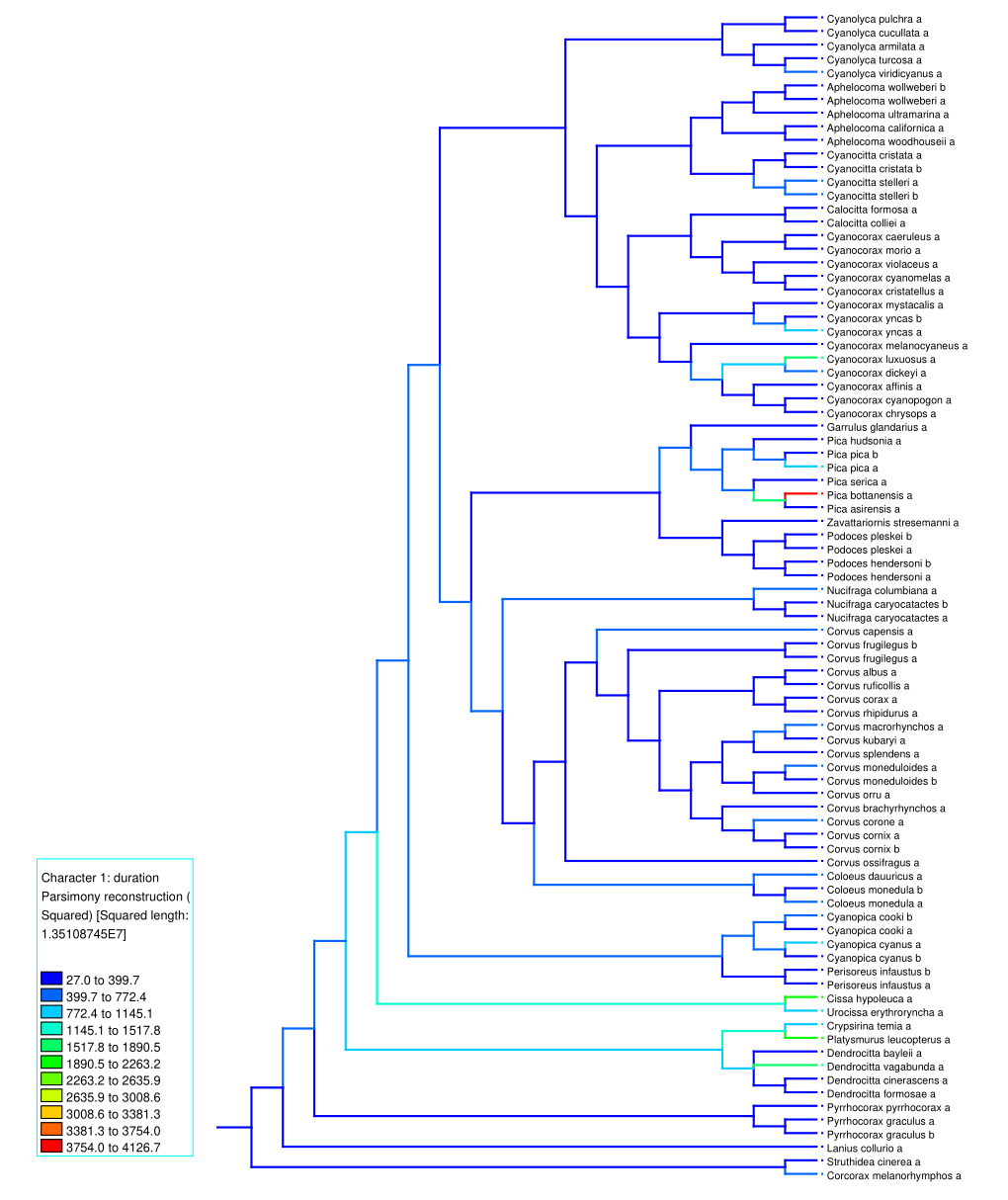

Supplement: Supplementary file 1 — Supplementary file1 (TIFF 334 kb) [file 10071_2025_2000_MOESM1_ESM.tiff]

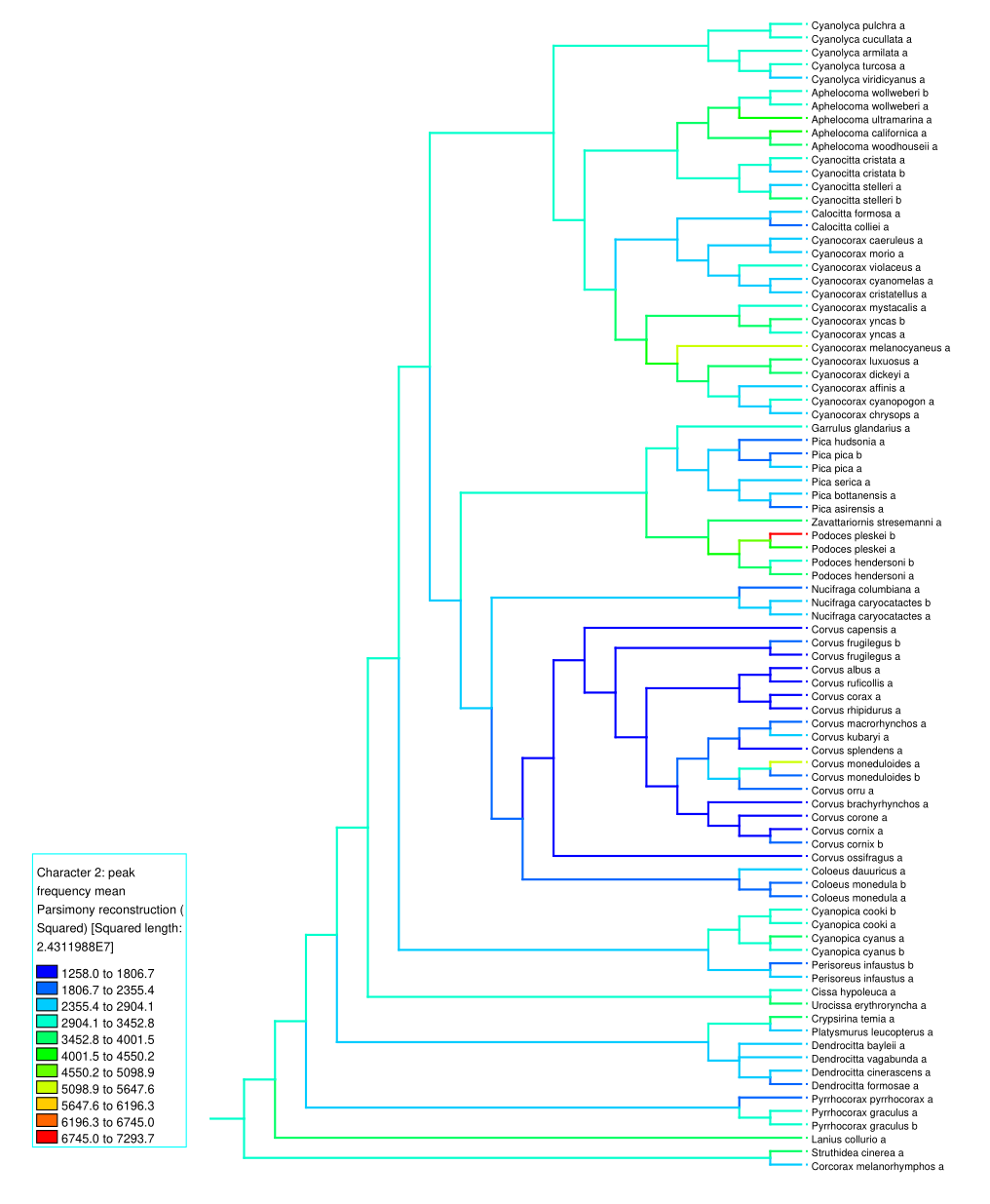

Supplement: Supplementary file 2 — Supplementary file2 (TIFF 343 kb) [file 10071_2025_2000_MOESM2_ESM.tiff]

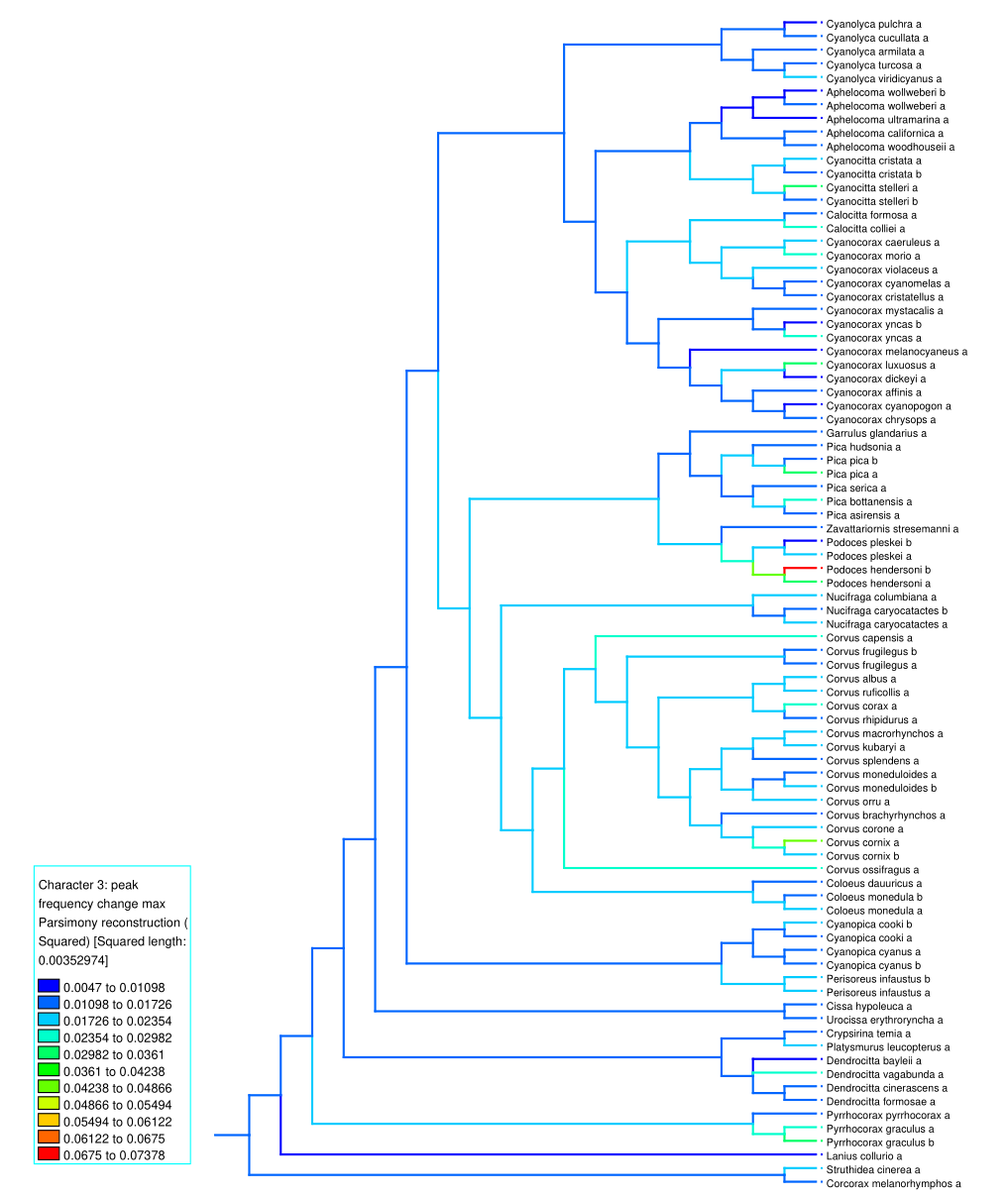

Supplement: Supplementary file 3 — Supplementary file3 (TIFF 343 kb) [file 10071_2025_2000_MOESM3_ESM.tiff]

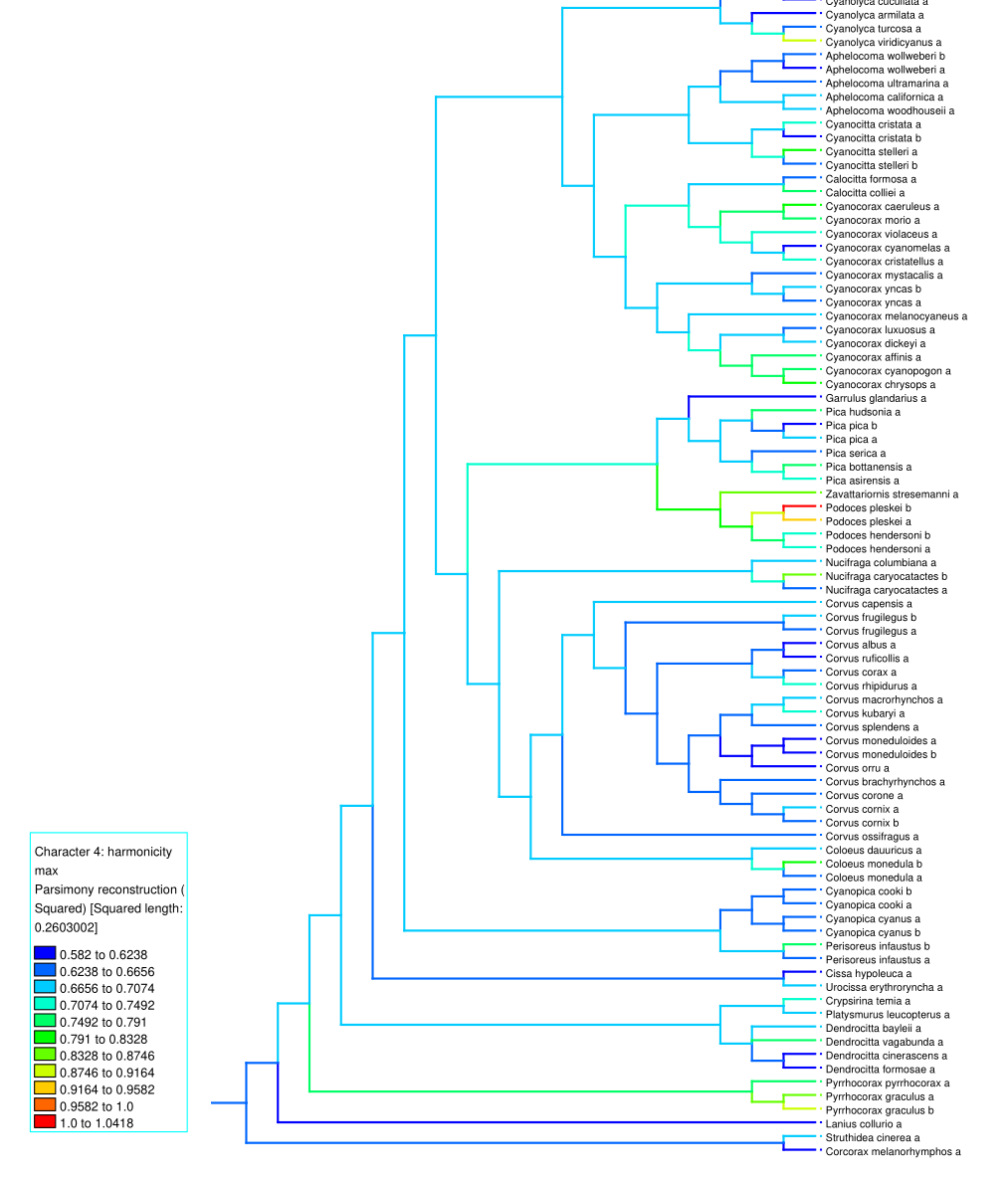

Supplement: Supplementary file 4 — Supplementary file4 (TIFF 333 kb) [file 10071_2025_2000_MOESM4_ESM.tiff]

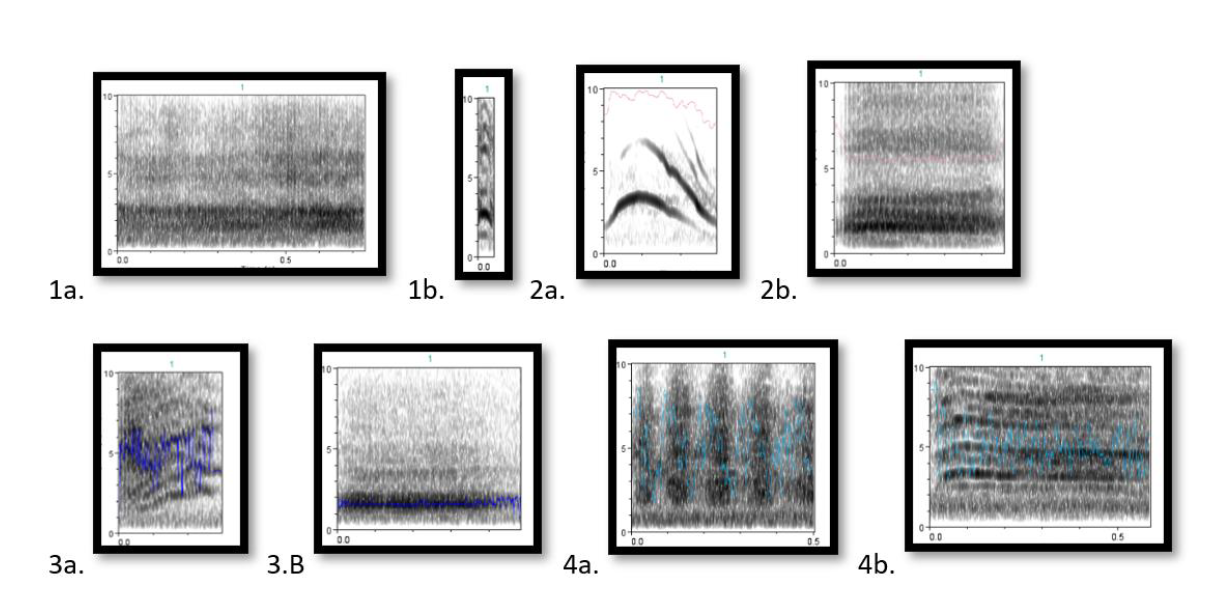

Supplement: Supplementary file 7 — Supplementary file7 (PNG 372 kb) [file 10071_2025_2000_MOESM7_ESM.png]
